# Can trophectoderm RNA analysis predict human blastocyst competency?

**DOI:** 10.1080/19396368.2019.1625085

**Published:** 2019-06-27

**Authors:** Panagiotis Ntostis, Georgia Kokkali, David Iles, John Huntriss, Maria Tzetis, Helen Picton, Konstantinos Pantos, David Miller

**Affiliations:** a Department of Discovery and Translational Science, LICAMM, University of Leeds, Leeds, UK; b Department of Medical Genetics, Medical School, National and Kapodistrian University of Athens, Athens, Greece; c Genesis Athens hospital, Reproductive medicine Unit, Athens, Greece

**Keywords:** Transcriptome, RNA sequencing, differential gene expression, trophectoderm, implantation competence

## Abstract

A systematic review of the literature showed that trophectoderm biopsy could assist in the selection of healthy embryos for uterine transfer without affecting implantation rates. However, previous studies attempting to establish the relationship between trophectoderm gene expression profiles and implantation competency using either microarrays or RNA sequencing strategies, were not sufficiently optimized to handle the exceptionally low RNA inputs available from biopsied material. In this pilot study, we report that differential gene expression in human trophectoderm biopsies assayed by an ultra-sensitive next generation RNA sequencing strategy could predict blastocyst implantation competence. RNA expression profiles from isolated human trophectoderm cells were analysed with established clinical pregnancy being the primary endpoint. Following RNA sequencing, a total of 47 transcripts were found to be significantly differentially expressed between the trophectoderm cells from successfully implanted (competent) versus unsuccessful (incompetent) blastocysts. Of these, 36 transcripts were significantly down-regulated in the incompetent blastocysts, including Hydroxysteroid 17-Beta Dehydrogenase 1 (*HSD17B1*) and Cytochrome P450 Family 11 Subfamily A Member 1 (*CYP11A1*), while the remaining 11 transcripts were significantly up-regulated, including *BCL2* Antagonist/Killer 1 (*BAK1*) and KH Domain Containing 1 Pseudogene 1 (*KHDC1P1*) of which the latter was always detected in the incompetent and absent in all competent blastocysts. Ontological analysis of differentially expressed RNAs revealed pathways involved in steroidogenic processes with high confidence. Novel differentially expressed transcripts were also noted by reference to a de novo sequence assembly. The selection of the blastocyst with the best potential to support full-term pregnancy following single embryo transfer could reduce the need for multiple treatment cycles and embryo transfers. The main limitation was the low sample size (N = 8). Despite this shortcoming, the pilot suggests that trophectoderm biopsy could assist with the selection of healthy embryos for embryo transfer. A larger cohort of samples is needed to confirm these findings.

**Abbreviations:** AMA: advanced maternal age; ART: assisted reproductive technology; CP: clinical pregnancy; DE: differential expression; FDR: false discovery rate; IVF: in vitro fertilization; LD PCR: long distance PCR; qRT-PCR: quantitative real-time PCR; SET: single embryo transfer; TE: trophectoderm

## Contextual review of the literature

### Success and failure in assisted reproduction technology (ART)

The rapid expansion in the use of ART since its origins in the late 1970s (Steptoe and Edwards 1978) has been accompanied by gradual improvements in pregnancy and delivery rates. For in vitro fertilization (IVF) cycles in 1997, the clinical pregnancy (CP) rate per embryo transfer was 26.1%, with a delivery rate of 20.9%. The corresponding figures for intra-cytoplasmic sperm injection (ICSI) cycles were 26.4% and 21.5%, respectively (Nygren and Andersen 2001). In 2012, the CP and delivery rates per aspiration and per embryo transfer were 29.4% and 33.8%, respectively, with 27.8% and 32.3%, respectively, for ICSI cycles. The overall delivery rate per embryo transfer was 28.2% (Calhaz-Jorge et al. 2016). Among contemporary practitioners, the agreed consensus is that improvements in ART success rates are needed and that the best approach to achieve a successful pregnancy would be to identify the single most competent blastocyst for single embryo transfer (SET) within each IVF cycle.

Chromosomal abnormalities occurring in the oocyte or the embryo are regarded as the most frequent causes of early implantation failure (Munne et al. 1998; Raziel et al. 2002) and are strongly related to advancing maternal age (AMA) (Nagaoka et al. 2012; Franasiak et al. 2014). Lack of synchronization between embryonic development on the one hand and receptivity of the female uterus on the other, as well as morphological and other anomalies of the uterus can also lead to implantation failure (Achache and Revel 2006; Margalioth et al. 2006). Sperm quality is also linked to early embryo developmental defects (Loutradi et al. 2006) with molecular paternal factors such as sperm DNA and RNA integrity thought to affect embryo implantation and pregnancy outcome (Robinson et al. 2012; Sendler et al. 2013; Ntostis et al. 2017).

At the genomic level, biopsies together with preimplantation genetic screening (PGS) have been used for aneuploidy detection prior to embryo transfer. In this regard, blastocyst biopsy is considered a safer approach for the embryo than cleavage-stage biopsy (Scott et al. 2013). The considered impact of aneuploidy on implantation potential and successful pregnancy is contradictory and beyond the scope of this review. The reader is respectfully directed to the reviews of Harper and of Twisk (Twisk et al. 2006; Harper 2018) for more detailed background of the arguments surrounding the clinical utility of PGS. Suffice to say that some studies have shown a positive effect of aneuploidy screening on blastocyst implantation of advanced maternal age mothers (Lee et al. 2015), while others showed no such effect (Harton et al. 2013; Moayeri et al. 2016). Blastocyst morphology and developmental rates but not cleavage morphology have been significantly associated with euploidy (Majumdar et al. 2017). This study also reported higher implantation rates for good quality embryos (blastocysts with grade ≥3 and ICM/TE quality AA, AB, and BA (Gardner and Schoolcraft 1999) and cleavage stage (Day-3) embryos consisting of 7–9 cells, with no fragmentation and stage-specific cell sizes, were considered of good quality compared with those of poorer quality, while similar implantation rates were found in all euploid blastocysts regardless of morphology. Nevertheless, in combination with more recent approaches (see below), morphology assessment could help improve the selection of more competent embryos (Gardner and Balaban 2016).

### Morpho-kinetics and time lapse imaging

Considering that SET has well-established benefits to both the mother and the fetus (De Neubourg and Gerris 2003; Bergh 2005), determining the best embryo for transfer should be a priority for IVF treatment. In brief, several criteria have been applied for this purpose including visual, metabolomic and morphokinetic (time-lapse) evaluation (Vergouw et al. 2008; Meseguer et al. 2012; Gardner and Balaban 2016). Real time, time-lapse imaging has been used for this purpose in ART for different species (Holm et al. 2002; Pribenszky et al. 2010) including human (Payne et al. 1997; Van Blerkom et al. 2001; Meseguer et al. 2012). Morphological criteria have been established, that help improve implantation and pregnancy rates; however, these rates have remained relatively unchanged in recent years (De Geyter et al. 2018). A combination of time-lapse imaging with aneuploidy detection screening led to a reported increase in the selection efficiency of competent blastocysts for transfer (Yang et al. 2014).

Wong and colleagues developed a real-time imaging algorithm for the determination of candidate embryos that could successfully reach the blastocyst stage. Three different parameters were employed, including the duration of first cleavage, the time between first and second mitotic division and between the second and third mitosis (Wong et al. 2010). Meseguer and colleagues’ equivalent algorithm (Meseguer et al. 2011) is based on morphokinetic criteria classifying the embryos into ten different classes, based on the timing of mitotic divisions combined with the time between intracytoplasmic sperm injection (ICSI) and the formation of five cell embryos. Negative predictors were also considered, such as the occurrence of multiple nuclei in four-cell embryos, the different size of the two blastomeres at the cleavage stage and cleavage directly from one to three blastomere embryos.

As indicated above, time-lapse and morphokinetic criteria are promising developments for improving implantation and pregnancy rates but several issues remain, including the possibility that imaging may be misleading (Herrero and Meseguer 2013). Moreover, the time ranges specified by the various algorithms might not represent true embryo development due to a lack of consideration of embryonic gene activation (EGA) that occurs mainly between the 4 and 8 cell stage. For these reasons, a better understanding of the relationship between morphokinetic criteria and successful pregnancy is necessary (Armstrong et al. 2015).

### Metabolomics of spent media and reproductive fluids

In a completely different approach, metabolomic profiles for certain biomarkers secreted into or absorbed by the embryo to or from the culture media have been reported to coincide with embryo quality (Seli et al. 2007; Vergouw et al. 2008). Different constituents such as lactate, albumin, glutamate, pyruvate and glucose are potentially detected in the culture media by this approach. A major limitation derives from the high levels of metabolic plasticity that occurs during early embryo development and the fact that embryo development and metabolism varies under different culture conditions (Krisher et al. 2015). Other limitations are linked to the variability of commercial culture media, as well as metabolite conversions that makes the determination of optimal metabolic profiles linked to successfully implanted blastocysts more difficult. Although they might play a role during early embryo development, current methods in metabolomics may be insufficiently sensitive to determine the quantities of particular metabolites (Krisher et al. 2015). Several biochemical markers have been investigated in this regard including matrix metalloproteinases MMP-2 and MMP-9 (Turpeenniemi-Hujanen et al. 1995) and Leukemia Inhibitory Factor (LIF; (Cullinan et al. 1996). Because of the low quantities of these molecules in culture media or aspirated uterine fluid, it has proved difficult to link LIF quantities and functions in the uterus with successful embryo implantation in the human (Kimber 2005).

High performance liquid chromatography has been used to correlate the turnover of three amino acids Asn, Gly, Leu with clinical pregnancy and live birth (Brison et al. 2004). Similarly, sHLA-G ELISA has been used for the determination of sHLA-G levels linked to developmentally competent embryos (Noci et al. 2005). Although promising, these approaches have still to consider the difficulties of their application on a daily/routine basis for assessing embryo competency. Alternative metabolomic approaches including nuclear magnetic resonance (NMR) and near-infrared spectroscopy (NIR) have not, to date, managed to successfully predict pregnancies (Hardarson et al. 2012; Kirkegaard et al. 2014).

### Gene expression profiling of trophectoderm biopsies

Gene expression in the cells of the blastocyst trophectoderm (TE), which go on to form the extra-embryonic tissues can be analysed with higher sensitivity and accuracy than was possible beforehand. Hence, molecular biological investigation of TE cells has the potential to become a useful predictive tool for implantation competence among embryos developed to the blastocyst stage. Two animal studies in bovine and mouse attempted to correlate TE RNA expression with blastocyst implantation potential. The first (El-Sayed et al. 2006) analyzed pooled samples from the bovine that due to blastocyst variability within the pools, may not have accurately reflected the implantation competency of individual blastocysts. The study reported 52 transcripts that were significantly differentially expressed and distinguished between pregnant and non-pregnant groups. RNAs relating to implantation, growth factors and signal transduction were significantly elevated in the pregnant group. The second study was conducted on single mouse blastocyst transfers with implantation success in 40 out of 70 blastocysts (60%), 28 of which developed to term. Two transcripts, UDP-GlcNAc:BetaGal Beta-1,3-N-Acetylglucosaminyltransferase 5 (B3gnt5), for glycolipid metabolism and Eomesodermin (Eomes) a transcriptome activator during embryo development, as well as various Wnt/β-catenin signaling members, showed significant down-regulation in non-implanted blastocysts (Parks et al. 2011).

Two studies in humans attempted to link specific TE RNA profiles and implantation potential following embryo-transfer. The studies employed either multiple (Jones et al. 2008) or single (Kirkegaard et al. 2015) blastocyst transfers with two possible outcomes recorded (implantation success or failure) per blastocyst transfer. The first study used a microarray-based approach to examine gene expression in 153 samples from either single or pooled blastocyst biopsies. No specific transcripts were reported; rather, wider ontological descriptions that accompanied competent and incompetent blastocyst implantation were described. While encouraging, this report did not disclose the subsequent signature of competent versus incompetent blastocysts. The second study used an RNA sequencing approach on single blastocyst biopsies. Here, full term delivery was the primary endpoint. The limitations of the approach used in this study will be discussed later (see Discussion).

Data for the pilot work reported herein was derived from 8 human blastocyst samples and employed an ultra-sensitive single-cell RNA sequencing approach to deal with the low TE cell numbers available and consequently the exceptionally low RNA quantities recoverable. As part of the bioinformatics pipeline, a bespoke, *de novo* transcript assembly analysis was also developed to help reveal potential novel transcripts associated with implantation competency. Considering that blastocyst biopsy does not adversely affect developmental competency nor embryonic implantation (Jones et al. 2008; Scott et al. 2013; Cimadomo et al. 2016), the study’s aim was to test the hypothesis that high sensitive RNA-seq of blastocyst biopsies could distinguish between implantation competent and incompetent blastocysts. Further development of the protocol could lead to the selection of the single most viable blastocysts for transfer, leading to more successful treatment outcomes.

## Results

### Clinical samples and outcomes

As part of their IVF treatment, five patients consented to and underwent blastocyst biopsy prior to uterine transfer (please see Materials and Μethods supplementary file for details). Clinical pregnancy was established in three patients (competent group), where all blastocysts implanted resulting in one set of non-identical twins and two singleton term pregnancies. Two patients (incompetent group) failed to achieve successful implantation following the transfer of two embryos each. Gene expression profiles from pre-transfer TE biopsies (six to eight aspirated cells in each case) were obtained and compared from all eight transferred blastocysts, without prior knowledge of their pregnancy status. Each group included two good quality hatching blastocysts (Gardner and Schoolcraft 1999). The implanted group involved two expanded (4BB) blastocysts and the non-implanted group one expanded (4AB) and one expanding (3AB) blastocyst (Gardner and Schoolcraft 1999). Full details on the bioethics approvals and blastocyst biopsy can be found in Materials and Methods supplementary file.

### Transcriptome characterization and ontological analysis

Our RNA sequencing strategy depended on a high sensitivity approach (see Materials and Methods supplementary file for details) and detected over 10,000 TE transcripts that exceeded 1 count per million reads (CPM; Supplemental Table 1). The number of average mapped correctly paired reads for transcripts in TE cells of competent blastocysts was 20.5 ± 2 million compared with 21 ± 1 million for the incompetent blastocysts. The top biological processes for these transcripts indicated a highly specialised gene expression profile with significantly low false discovery rates (FDRs) (Supplemental Table 2). Cell adhesion and DNA repair were among the top biological processes alongside cell division, mitotic nuclear division and DNA replication, highlighting high levels of the cellular proliferation that is a feature of early embryonic development. Several transcripts involved in DNA repair processes were also reported with transcription-coupled nucleotide-excision repair, double-strand break repair via recombination and base-excision repair being particularly noteworthy and also indicating rapid cellular proliferation (Gilbert 2000).

Post-transcriptional regulatory processes revealed by the analysis included mRNA splicing and export from the nucleus, mRNA catabolic processing and regulation of mRNA stability, illustrating the central importance of these functions during early embryo development. Translation and post-translational ontologies with very low FDR levels were also detected. These processes included protein transport, polyubiquitination, sumoylation, folding and extensive post-translation modification, including K11 and K48-linked ubiquitination. Metabolism and energy-oriented bioprocesses involving oxidative phosphorylation were highlighted demonstrating high rates of metabolic activity in developing embryos.

### RNA characterization and exonic representation of differentially expressed transcripts

**Figure 1.**
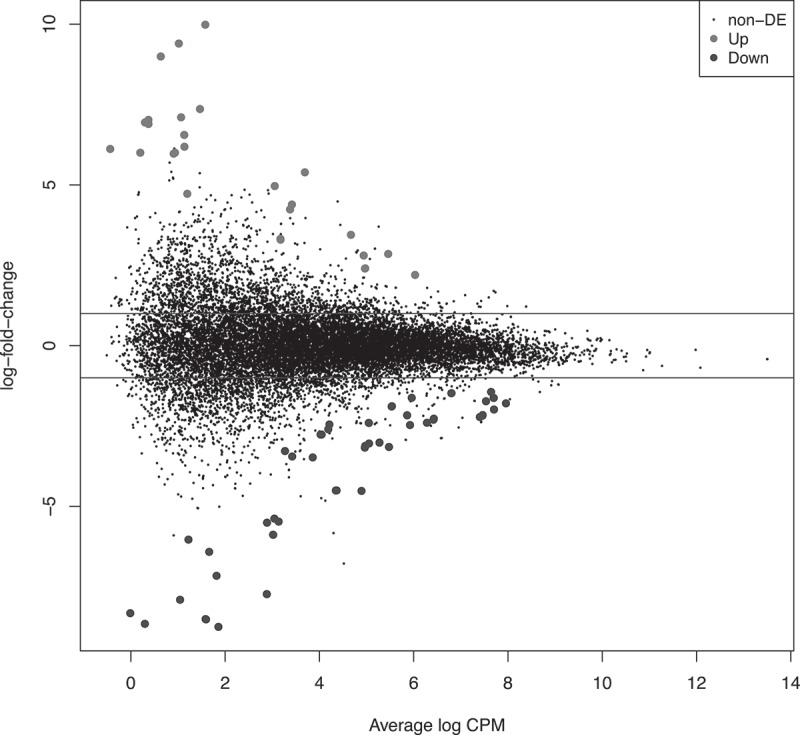
Scatter plot of DE (successful vs unsuccessful blastocyst) derived by edgeR analysis. Results are from edgeR analysis with the Y-axis representing the log_2_ fold change (log-FC) and the X axis the average log counts-per-million reads (CPM). Upper and lower horizontal lines represent a log-FC of 1 and −1 up and down regulation, respectively, in incompetent blastocysts. Significantly up (light grey)- and down (dark grey)-regulated transcripts are indicated. There were 73 DE transcripts overall representing known genes (47) with the remainder being a mix of alternative splice variants and predicted transcripts.

The edgeR exact test was used to reveal significantly differentially expressed (DE) transcripts between competent and incompetent blastocysts (Figure 1; please see Μaterials and Μethods supplementary file for full details). The DE transcripts belonged to 47 unique genes (Table 1). Given that the competent group represents normal TE expression levels, the current study focused on the significantly lower/higher expression levels of the incompetent blastocysts. Following normalization of the RNA sequencing results, 36 transcripts were found to be significantly down-regulated in these blastocysts with the remainder being up-regulated (FDR < 0.05). These included KH Domain Containing 1 Pseudogene 1 (*KHDC1P1*), the apoptosis regulator *BCL2* Antagonist/Killer 1 (*BAK1*), suggesting a potentially significant regulatory function perhaps relating to an underlying apoptotic process. All DE transcripts had at least three-fold-change, apart from *KH RNA Binding Domain Containing, Signal Transduction Associated 3 (KHDRBS3)* and Emopamil Binding Protein (Sterol Isomerase; *EBP*) that were slightly lower at 2.7-fold.

**Table 1.** Differentially expressed (DE) transcripts between competent and incompetent blastocysts.

AA	Gene Name	log FC	log CPM	p value	FDR	AA	Gene Name	log FC	log CPM	p value	FDR
1	*EPHA1*	−8.65	0.30	1.10E-05	8.46E-03	25	*C14orf1*	−2.16	5.86	3.77E-05	1.85E-02
2	*CD274*	−8.51	1.59	7.93E-07	1.45E-03	26	*IDI1*	−2.15	7.46	1.34E-04	3.72E-02
3	*LYPD3*	−7.73	2.88	3.44E-11	1.79E-07	27	*FABP3*	−1.98	7.70	5.50E-06	5.49E-03
4	*HSPB8*	−7.16	1.82	6.32E-06	5.49E-03	28	*ZCCHC17*	−1.98	7.70	4.78E-06	5.49E-03
5	*PLEKHA6*	−6.03	1.22	2.34E-04	4.98E-02	29	*ASAH1*	−1.89	5.53	2.06E-04	4.83E-02
6	*TMPRSS2*	−5.88	3.02	4.66E-10	1.69E-06	30	*LOC 101,929,066*	−1.88	5.54	1.98E-04	4.81E-02
7	*DUSP6*	−5.47	3.14	1.22E-04	3.59E-02	31	*FDPS*	−1.79	7.96	1.78E-04	4.60E-02
8	*ACSS2*	−4.52	4.89	9.06E-05	3.20E-02	32	*TPM4*	−1.73	7.53	1.26E-04	3.63E-02
9	*CYP11A1*	−4.50	4.37	7.49E-05	2.97E-02	33	*DBI*	−1.62	7.70	8.54E-05	3.18E-02
10	*CYP51A1-AS1*	−3.47	3.86	4.04E-05	1.85E-02	34	*IFT20*	−1.62	5.96	7.00E-05	2.86E-02
11	*SC5D*	−3.44	3.42	1.01E-04	3.51E-02	35	*KHDRBS3*	−1.48	6.80	1.15E-04	3.57E-02
12	*DHCR7*	−3.15	5.48	4.06E-05	1.85E-02	36	*EBP*	−1.44	7.64	1.21E-04	3.59E-02
13	*CD24*	−3.12	4.97	1.89E-04	4.81E-02	37	*ADCK1*	2.20	6.03	2.20E-04	4.83E-02
14	*CYP51A1*	−3.04	5.05	1.58E-05	1.05E-02	38	*CHST2*	2.41	4.97	2.03E-04	4.83E-02
15	*INSIG1*	−3.02	5.27	6.66E-07	1.42E-03	39	*BAK1*	2.86	5.46	1.65E-05	1.05E-02
16	*MIR548XHG*	−2.76	4.02	1.96E-04	4.81E-02	40	*PLLP*	3.45	4.67	1.16E-04	3.57E-02
17	*ARHGAP10*	−2.60	4.19	2.06E-04	4.83E-02	41	*FAM171A1*	4.97	3.05	4.59E-05	1.95E-02
18	*MVD*	−2.47	5.92	1.37E-05	9.83E-03	42	*KHDC1P1AS*	5.39	3.69	1.07E-11	1.12E-07
19	*NR3C2*	−2.45	4.21	2.20E-04	4.83E-02	43	*KHDC1P1*	5.39	3.69	1.07E-11	1.12E-07
20	*HSD17B1*	−2.40	5.05	3.63E-05	1.85E-02	44	*ZNF285*	6.00	0.94	7.97E-05	3.08E-02
21	*ABCG2*	−2.40	6.28	6.26E-06	5.49E-03	45	*KIAA0226L*	6.01	0.20	2.17E-04	4.83E-02
22	*SQLE*	−2.27	6.43	2.35E-08	6.13E-05	46	*DEFB124*	6.19	1.14	2.00E-05	1.22E-02
23	*MSMO1*	−2.22	7.40	5.15E-06	5.49E-03	47	*WNK3*	9.40	1.02	3.07E-06	4.26E-03
24	*IDI2-AS1*	−2.18	7.46	1.14E-04	3.57E-02						

The list shows 47 DE genes/transcripts that were highlighted by EdgeR analysis with 36 down-regulated (log FC < 0) and 11 up-regulated (log FC > 0) in incompetent blastocysts. Log FC is logarithm fold-change and log CPM is logarithm copies per million. The FDR was below 0.05 in all displayed transcripts.

**Figure 2.**
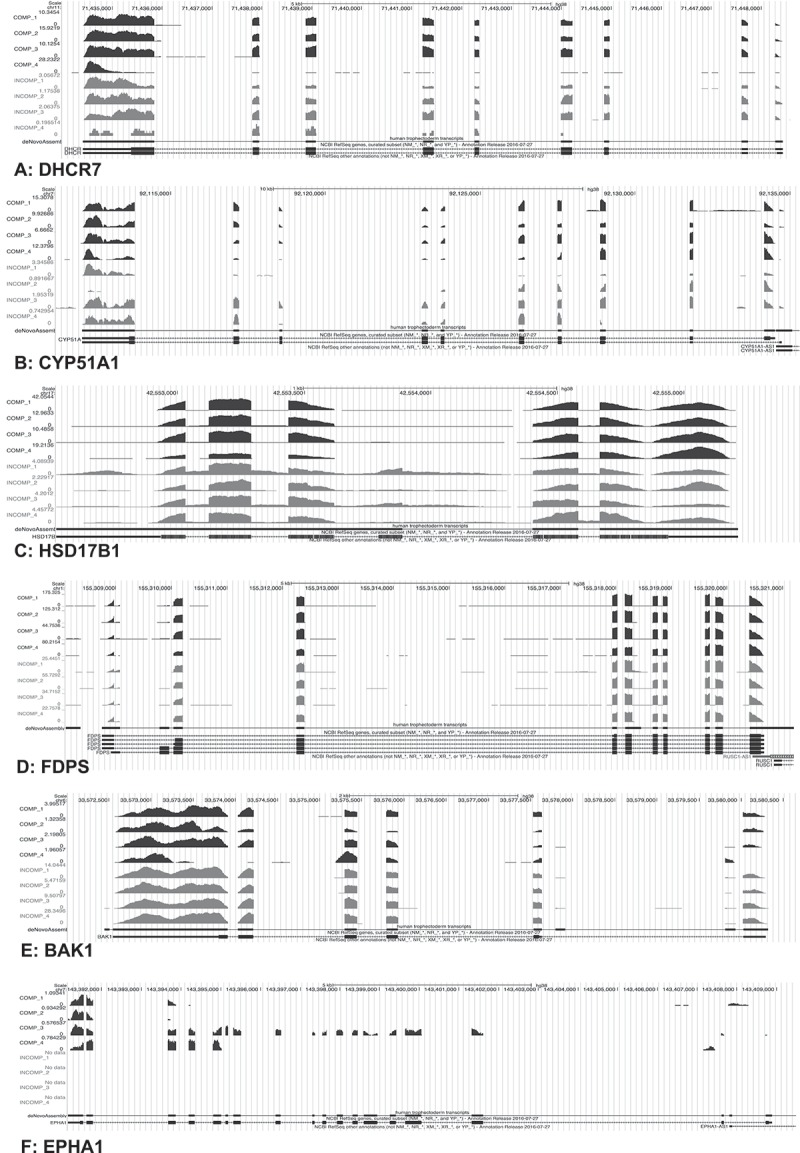
UCSC genome browser tracks of selected DE transcripts. The plot shows pile-ups of sequencing reads assembled into transcripts for six different genes in eight TE samples. The top four pile-ups (per gene) correspond to gene expression in the competent (COMP) blastocysts and the bottom four to the incompetent (INCOMP) blastocysts. A *de novo* transcriptome assembly track is shown below each pile-up guided by the hg38 reference genome (black). The track corresponds to all exons detected by StringTie, including novel exons. The bottom track per gene represents the NCBI RefSeq gene annotation. The boxes in the lower two pile-ups represent the exons and the arrows the introns and expected direction of gene expression. The graph depicts a total of four representative transcripts; *DHCR7* (A), *CYP51A1* (B), *HSD17B1* (C), and *FDPS* (D) that were down-regulated and one transcript, *BAK1* (E), that was up-regulated in incompetent blastocysts and one transcript, *EPHA1* (F), with poorer exon coverage. Scales for peak heights, representing pile-ups of read counts (CPM) are shown to the left of each plot.

With the exception of *CD274* (or *B7H1*), EPH Receptor A1 (*EPHA1*), RUN and Cysteine Rich Domain Containing Beclin 1 Interacting Protein Like (*RUBCNL)*, WNK- Lysine Deficient Protein Kinase 3 (*WNK3*) and Zinc Finger 285 (*ZNF285*), there was excellent coverage across all exons of the DE transcripts (Fujii et al. 2006; Holets et al. 2009; Iqbal et al. 2014). Figure 2 shows examples of down-regulated (A-D), up-regulated (E), and poor exonic coverage genes in incompetent blastocysts (F). More specifically, the transcripts 7-Dehydrocholesterol Reductase (*DHCR7*), Cytochrome P450 Family 51 Subfamily A Member 1 *(CYP51A1*), Hydroxysteroid 17-Beta Dehydrogenase 1 (*HSD17B1*) and Farnesyl Diphosphate Synthase (*FDPS*) showed good exonic coverage and were significantly down-regulated in incompetent blastocysts (Koo et al. 2010; Clemente et al. 2011; Assou et al. 2012; Iqbal et al. 2014). On the other hand, *BAK1* is an example of transcripts with good exonic representation that was more highly expressed in incompetent blastocysts. Novel and alternatively spliced exons are not unusual in genes expressed in early embryonic development (Revil et al. 2010). Our *de novo* assembly annotation approach, indicated exons additional to those present in the RefSeq annotation, with Cytochrome P450 Family 11 Subfamily A Member 1 (*CYP11A1*) and AarF Domain Containing Kinase 1 (*ADCK1*) among them (Supplemental Figure 1). *CYP11A1*, for example, showed additional exons that probably belong to an alternatively spliced transcript lying between the first and second exons (first intron) of the two gene isoforms shown (A). Similarly, additional exons were revealed in *ADCK1* between the first and the second (first intron) and third and fourth (third intron) exons, probably corresponding to alternatively spliced transcripts.

### Ontology and KEGG pathway analysis of the differentially expressed transcripts

**Figure 3.**
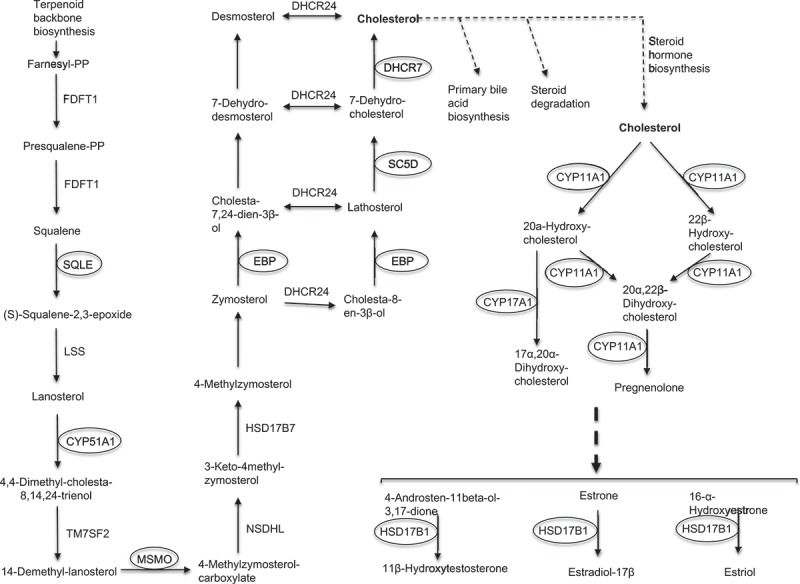
KEGG pathway for steroid/hormone biosynthesis with relevant DE transcripts. The background diagram was adapted from Kanehisa labs (KEGG database) and includes steroid biosynthesis (map00100 pathway) and steroid hormone biosynthesis (map00140 pathway) (Kanehisa and Goto 2000). The diagram shows eight highlighted genes with significantly down-regulated transcripts in incompetent blastocysts.

Functional annotation clustering of down-regulated RNAs in incompetent blastocysts was undertaken using the DAVID bioinformatics resources v6.8 (please see Μaterials and Μethods supplementary file for full details), revealing 4 clusters with enrichment scores > 2.0 (3.11 to 8.53). The first cluster (enrichment score: 8.53) included ontological terms involved mainly in steroid and sterol biosynthesis and metabolism, as well as oxidation-reduction (Supplemental Table 3). The second and third clusters with enrichment scores 5.79 and 5.19, involved steroid and cholesterol biosynthesis, respectively. The fourth cluster with enrichment score 3.11, also involved steroid biosynthesis, oxidation-reduction and nicotinamide adenine dinucleotide phosphate (NADP) metabolism (Supplemental Table 3). In contrast, transcripts more highly expressed in incompetent blastocysts, showed low enrichment scores (≤0.4) for terms including integral membrane component and transmembrane region that were not significant. Eight of the 36 down-regulated transcripts encode enzymes involved in cholesterol biosynthesis, including *EBP*, Sterol-C5-Desaturase (SC5D), 7-Dehydrocholesterol Reductase (*DHCR7*), and other transcripts important in steroidogenesis, such as *CYP11A1* and *HSD17B1* (Figure 3).

### Sequencing validation

**Figure 4.**
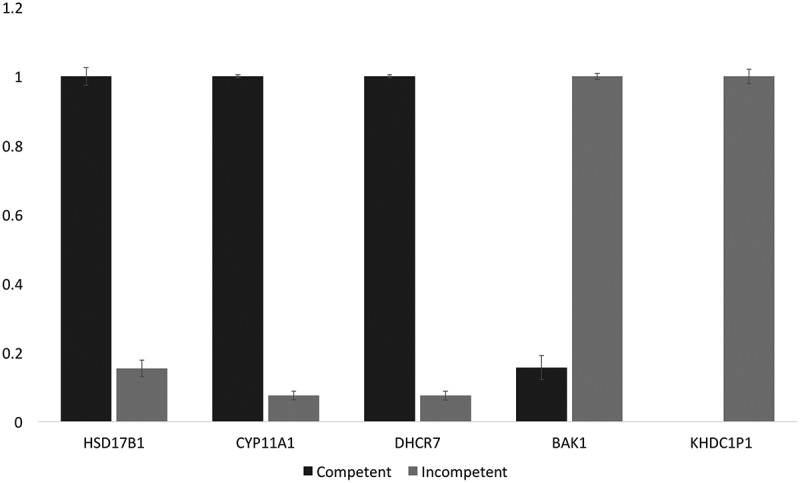
Normalized qPCR results for verification of DE analysis. Following 2^−ΔΔCt^ normalization and calculation relative to *GAPDH* expression (stable among our samples), the more highly expressed of the pairs in each case were set to 1.0 (arbitrary units). The chart shows that among the incompetent blastocysts, *HSD17B1, CYP11A1,* and *DHCR7* were significantly down-regulated, while *BAK1* and *KHDC1P1* were significantly up-regulated. Standard deviation error bars are also displayed in the graph. These results concord with the edgeR differential expression analysis obtained from the RNA-seq data.

Quantitative Real Time-PCR (see materials and methods supplementary file for details) was employed to validate the RNA sequencing results of the down-regulated (*HSD17B1, CYP11A1, DHCR7*) and up-regulated (*BAK1* and *KHDC1P1*) transcripts in the incompetent blastocysts with reference to *GAPDH* (Figure 4). Setting the more highly expressed mean 2^−ΔΔCt^ values for each comparison (competent versus incompetent) to 1.0 indicated complete agreement with the Edge R, RNA-seq differential expression analysis.

### Aneuploidy analysis from deep RNA sequencing

To ensure that implantation potential had not been compromised by possible chromosomal aneuploidies in the experimental samples, RNA sequencing data was used to conduct an *in silico* karyotype analysis (see Materials and Methods for full details, including addressing the SNP validation of blastocyst ploidy). The analysis supports the assumption that at the time of transfer, all embryos were euploid. Supplementary Figure 2 shows examples from incompetent blastocysts and all results are available at https://github.com/daveiles/human_TEbiopsy_eSNPanalysis.

## Discussion

An ultra-sensitive RNA-Seq approach employed the SmartSeq Ultra Low Input RNA kit (Takara-Clontech) combined with Nextera library preparation approach (Illumina), and was used to examine TE gene expression profiles of 6–8 cells biopsied from eight, Day-5 blastocysts that either went on (competent) or failed (incompetent) to support successful implantation (see supplemental material and methods). Although the same gynecologist conducted the embryo-transfers and a sensitive RNA sequencing approach was employed throughout, the low blastocyst and sample size were the main limitations of this study. Moreover, while every effort was made to select only idiopathically infertile patients, we cannot discount the possibility that endometrial receptivity and/or other maternal conditions not obvious with gynecological examination could have led to implantation failure independently of blastocyst competence. While the sample size was small, given the difficult recruitment of these samples and the fact that a more optimized RNA sequencing approach was employed compared with previous studies, it is worth reporting our results nonetheless.

An initial survey of the gene expression landscape of biopsied TE cells revealed a ‘signature’ enriched for ontologies involved in gene expression, cell adhesion, cell division, mitosis and apoptosis. These are all important biological processes occurring during early pre-implantation embryonic development (Brison 2000; Niakan et al. 2012). In addition, post-transcriptional and post-translational levels of gene expression regulation important during early embryogenesis (Braude 1979), were over-represented in the TE expression data. EdgeR analysis flagged a total of 47 significantly differentially expressed genes in incompetent blastocysts of which 36 and 11, respectively, were either down or up-regulated. The data on aneuploidy screening suggests that chromosomal imbalance did not confound our interpretation of the results of this pilot study. Embryo implantation was determined on Day 16 following oocyte retrieval, using serum beta-hCG; week 4 of gestation that was determined by ultrasound that demonstrated gestational sac; and beyond 7 weeks of gestation through the determination of fetal heartbeat. All four non-implanted blastocysts showed no measurements for the aforementioned parameters, indicating no sign of implantation.

The steroidogenic relationship among the 36 significantly down-regulated transcripts in incompetent blastocysts was one of the more intriguing results of this study. Steroid production and secretion by the conceptus are important steps for embryo-maternal recognition during implantation and subsequent placentation (Pusateri et al. 1990; Geisert and Yelich 1996). Endometrial receptivity depends on the interplay between the steroid hormones, including estradiol (E2), and progesterone (Bagchi et al. 2001). Steroid hormones classically act through nuclear receptors that regulate gene expression (Ing 2005). Estradiol promotes proliferation of endometrial cells, while progesterone increases endometrial secretion, promoting embryo-endometrial attachment. Estradiol treatment significantly increases the proliferation of TE cells (Wilmoth et al. 2010). Apart from promoting the embryo-maternal recognition, conceptus-derived estrogens alter the expression of endometrial genes related to immunity, production and transport of prostaglandin and growth factors (Ham et al. 1975; Ka et al. 2018), with both estrogens and progesterone altering the expression of growth factors and cytokines facilitating uterine receptivity (Dickmann et al. 1975; Shemesh et al. 1979; Niakan et al. 2012; Matsumoto 2017). During this pre-implantation period, molecular communication between TE and endometrial cells, includes the up-regulation of cytokines by the former and certain adhesion molecules, such as CD44, by the latter (Haouzi et al. 2011). Their down-regulation or loss could compromise the chances of successful embryo implantation. With the exception of one of the incompetent blastocysts where *CYP11A1* expression was almost undetectable, both *HSD17B1* and *CYP11A1* were present and significantly differentially expressed between the 2 groups, indicating their likely importance even at low levels. *HSD17B1* is associated with the reduction of estrogen and androgen inactivation, as part of steroid biosynthesis (Maglott et al. 2005) and has high expression levels in the placenta (Fagerberg et al. 2014). The gene has also been associated with recurrent miscarriage (Ntostis et al. 2015), endometriosis (Lamp et al. 2011) and with breast and endometrial cancer (Feigelson et al. 2006; Ashton et al. 2010). An early step in steroid biosynthesis involves the conversion of cholesterol to pregnenolone by CYP11A. A potential deregulation of this transcript could affect the production of a wide range of hormones and perhaps endometrium-embryo communication. It may also play a role in polycystic ovary syndrome (PCOS) and endometrial cancer risk (Ashton et al. 2010; Yu et al. 2014). Earlier comparisons between the inner cell mass (ICM) and TE gene expression in the bovine and mouse revealed significantly higher expression of RNAs for steroidogenic enzymes, including HSD3B1, HSD17B1, CYP11A1 and CYP19A1 in the TE (Houghton 2006; Ozawa et al. 2012; Hosseini et al. 2015), while steroid biosynthesis was significantly more highly expressed in the TE, compared with day-3 embryos (Assou et al. 2012).

In relation to earlier studies, other significantly down-regulated transcripts in incompetent blastocysts were also noteworthy. ATP Binding Cassette Subfamily G Member 2 (*ABCG2*), for example, a member of the ATP-transporting superfamily involved in drug resistance is highly expressed in normal TE (Janvilisri et al. 2005; Hardwick et al. 2007). *ABCG2* is possibly involved in protecting the developing embryo from toxic metabolites (Pal et al. 2007). LY6/PLAUR Domain Containing 3 (*LYPD3*), a gene involved in the post-translational modification of GPI-anchored proteins, was among the most up-regulated transcripts in patients suffering recurrent miscarriage (Choi et al. 2016). Expression of the pluripotency marker phosphatidylinositol-linked sialoglycoprotein *CD24* was increased from the earliest pre-implantation to the later post-implantation stages following embryonic genome activation (Shakiba et al. 2015). Fatty Acid Binding Protein 3 (*FABP3*) a transcript involved in acyl-coA esters and fatty acid intracellular transport was also down regulated in incompetent blastocysts. This gene is normally highly expressed in TE of human and mouse blastocysts (Blakeley et al. 2015). Finally, the Heart And Neural Crest Derivatives Expressed 1 (*HAND1*) transcription factor is involved in early trophoblast differentiation (Riley et al. 1998). Significant down-regulation of this gene in incompetent blastocysts suggests a potential role during the implantation process or early embryogenesis. Epigenetic regulation may also have played a role in the differential expression analysis of certain transcripts. Inappropriate epigenetic regulation of *ABCG2* in the incompetent blastocysts might have led to susceptibility to xenobiotic exposure (Babu et al. 2012). Moreover, steroid hormones are active in certain tissues (i.e., trophectoderm) through steroid nuclear receptors, including members of the *NR3A* and *NR3C* gene families. Together with other transcription factors, these steroid biosynthesis-related receptors could be epigenetically regulated (Martinez-Arguelles and Papadopoulos 2010). The current study revealed that expression of NR3C2 was significantly lower in the incompetent blastocysts.

The processed pseudogene *KHDC1P1* was one of the few significantly up-regulated transcripts found in incompetent blastocysts that was almost completely absent in competent blastocysts (Supplemental Figure 1). The gene is located in an unstable and rapidly evolving genomic region in eutherian mammals that includes Oocyte Expressed Protein (*OOEP*), Developmental Pluripotency Associated (*DPPA5*) and KH Domain Containing 3 Like, Subcortical Maternal Complex Member (*KHDC3L;* Pierre et al. 2007). Members of this gene family encode proteins that may be involved in RNA-binding (Cai et al. 2012) and at least one (*KHDC1A*), is potentially involved in apoptosis through global translation repression (Cai et al. 2012). Their exact function, however, is unclear. The high levels of expression of several members of this group, alongside *BAK1* (pro-apoptotic gene; (Chittenden et al. 1995) in incompetent blastocysts is particularly notable.

Several gene families with similar functions were shared with the Kirkegaard (*) study (Kirkegaard et al. 2015), including ATP binding cassette subfamilies (ABCG2, ABCB10*), cytochrome 450 family members, including CYP11A1, CYP51A1, CYP2F1*, and the hydroxysteroid dehydrogenases *HSD17B1* and HSD17B11*. A full list of shared gene families is available in Supplemental Table 4. Differences between their study and ours may have been due to a combination of factors, including the use of library construction not well suited to ultra-low initial RNA inputs. The absence of a *de novo* assembly step of the RNA sequencing data may also have impeded the detection of *de novo* assembled transcripts. Due to the relatively high variation reported for TE gene expression, the Kirkegaard study may also have lacked power to detect differentially expressed transcripts. We tried to avoid these pitfalls by applying a more evenly distributed, ultra-sensitive deep RNA sequencing approach and used more samples (8 in total) and only reported transcripts with differential fold-change above or close to 3.

A recent study involved the comparison between blastocysts from younger (< 35 yo) and older (≥ 35 yo) mothers (Kawai et al. 2018). The average younger and older maternal ages were, respectively, 33.0 years and 37.6 years. Results from this study were based on RNA isolated from whole blastocysts, which included inner cell mass (ICM). Considering that young patients are generally more fertile, we reasoned that data from our competent and their young blastocysts should have corresponded. In the current study, the average maternal age was 32.8 years for the competent and 35.0 years for the incompetent blastocysts and indeed, 8 differentially expressed transcripts were identical in both studies. *ADCK1* was up-regulated in both older mothers and our incompetent blastocysts, while Rho GTPase Activating Protein 10 (*ARHGAP10*) Intraflagellar Transport 20 (*IFT20*), Nuclear Receptor Subfamily 3 Group C Member 2 (*NR3C2*) and Zinc Finger CCHC-Type Containing 17 (*ZCCHC17*) were up-regulated in both young mothers and our competent blastocysts. *IFT20* is important for intracellular transport involved in protein trafficking, including recycling of immune signaling components (Follit et al. 2006). *NR3C2* encodes a nuclear hormone receptor that acts as a transcription factor binding to mineralocorticoid (aldosterone and glucocorticoids) response elements (MRE) and activates target genes raising water and ion transport activity (Arriza et al. 1987). Finally, *ZCCHC17* corresponds to a zinc-finger protein. The remaining 3 transcripts N-Acylsphingosine Amidohydrolase 1 (*ASAH1), CYP11A1* and Diazepam Binding Inhibitor, Acyl-CoA Binding Protein (*DBI*) are involved in cellular metabolism and were up-regulated in the competent blastocysts and blastocysts derived from the mature patients. This apparent discrepancy may have been due to technical reasons and/or sample differences.

## Conclusions

We used an ultra-sensitive RNA sequencing strategy to provide a more reliable selection of markers for determining blastocyst implantation competence. Our pilot study (together with other reports) suggests that TE biopsy can predict blastocyst competence and should be more extensively evaluated for this purpose. As TE biopsy does not appear to affect implantation rates, predicting blastocyst implantation competency could be a safe and reliable approach to take in conjunction with SET.
