# Supplementary material for: Can trophectoderm RNA analysis predict human blastocyst competency?
Source: Syst Biol Reprod Med. 2019 Jun 27;65(4):312–25. doi: 10.1080/19396368.2019.1625085 (PMC6816490; doi:10.1080/19396368.2019.1625085)
Supplement: Supplemental Material [file IAAN_A_1625085_SM9021.zip › 2018 336.r2 Supplemental Figure 1 title and details.docx]

**Supplemental Figure 1. Additional UCSC browser tracks of selected DE**.

The plots show additional pile-ups guided by hg38 human reference genome and RefSeq annotation for three transcripts, showing down-regulation of *CYP11A1* (**A**) and up-regulation of *ADCK1* (**B**) in incompetent (INCOMP) blastocysts, with virtual absence of *KHDC1P1* in the competent (COMP) blastocysts (**C**). Note the presence of additional exons shown on the StringTie but not RefSeq annotation corresponding with reads from the blastocyst biopsies, suggesting expression of novel, alternate splice variants.
