# Supplementary material for: Can trophectoderm RNA analysis predict human blastocyst competency?
Source: Syst Biol Reprod Med. 2019 Jun 27;65(4):312–25. doi: 10.1080/19396368.2019.1625085 (PMC6816490; doi:10.1080/19396368.2019.1625085)
Supplement: Supplemental Material [file IAAN_A_1625085_SM9021.zip › 2018 336.r2 Supplemental Figure 2 title and info.docx]

**Supplemental Figure 2. Detection of chromosomal aberrations using RNA-Seq data.**

eSNP karyotyping analysis from the RNA sequencing data for all TE biopsies was carried out (see Materials and Methods). Loss of heterozygosity (LOH) (**A**-**D**) and allelic ratio (**E**-**I**) plots for incompetent blastocysts are shown. Homozygous SNPs are shown in blue and heterozygous SNPs present or absent in the db151Common table are shown, respectively, in pink and red for each autosome (**A**-**D**). They show essentially that fully representative SNP coverage was obtained for all biopsies. The allelic-ratio plots are moving averages based on expected versus reported SNP sequences across the individual chromosomes (**E**-**H**), with a simulated trisomy 18 included (**I**). The scale to the right represents –log(10) p-values for significant fold change following Benjamini-correction as shown by bars with differential shading above the traces (the more significant the fold change, the darker the shade). In the simulated trisomy, the software has flagged the likely duplication of chromosome 18. Note that both the allelic ratios and SNP maps are required to indicate whether the anomaly is likely to be a duplication or a deletion. These tests indicated that all TE biopsies were euploid at the time of transfer.
