# Supplementary material for: Can trophectoderm RNA analysis predict human blastocyst competency?
Source: Syst Biol Reprod Med. 2019 Jun 27;65(4):312–25. doi: 10.1080/19396368.2019.1625085 (PMC6816490; doi:10.1080/19396368.2019.1625085)
Supplement: Supplemental Material [file IAAN_A_1625085_SM9021.zip › 2018 336.r2 Supplemental Figure 3 title and info.docx]

**Supplemental Figure 3. Agarose gel of PCR products derived from qPCR**.

The gel confirms the specificity of the designed primers. Following the ladder (lane 1), confirmatory qPCR products are shown for competent (even numbered lanes) and incompetent (odd numbered lanes) blastocysts in the following order. *GAPDH*, *HSD17B1,* *CYP11A1*, *DHCR7*, *BAK1* and *KHDC1P1* are displayed from left to right (lane 2-13). There was no amplification for *KHDC1P1* in competent blastocysts.
