# Supplementary material for: Can trophectoderm RNA analysis predict human blastocyst competency?
Source: Syst Biol Reprod Med. 2019 Jun 27;65(4):312–25. doi: 10.1080/19396368.2019.1625085 (PMC6816490; doi:10.1080/19396368.2019.1625085)
Supplement: Supplemental Material [file IAAN_A_1625085_SM9021.zip › Ntostis_etal_supplemental_Materials_and_Methods.docx]

**Materials and Methods**

1. Patient recruitment and ART treatment

Patients with idiopathic infertility treatment at the Reproductive Medicine Unit, Genesis Athens Clinic, Greece and scheduled to undergo fresh embryo transfer at the blastocyst stage (day 5) with no uterine abnormalities, were eligible to participate in this study.

2. Ethics approval and consent to participate

The study was approved by the National Health System, A’ Administration of the Health District of Attica, Greece, General Children Hospital “Aghia Sofia” (Reference – Protocol Number: 19964/04-09-2014), the Greek National Authority of Assisted Reproduction and Genesis Athens clinic. Patients participating in the study provided signed informed consent to TE biopsy before Day 5 transfer.

3. Εmbryo culture and blastocyst biopsy procedure

Oocytes were fertilized by intracytoplasmic sperm injection (ICSI) and cultured in continuous single culture media with 5% Human Serum Albumin (HSA) (IrvineScientific, CA, USA) to the blastocyst stage. At 120–150 h post-insemination, blastocysts were evaluated according to their degree of expansion and the quality of inner cell mass and TE cells (Balaban et al. 2011). Blastocysts selected for embryo-transfer were subjected to TE biopsy 2 hours before their transfer to the uterus. In brief, using a series of 2 to 3 pulses on a non-contact laser (Zilos-tk; Hamilton Thorne Biosciences, Beverley, AM, USA), a small hole in the zona pellucida was opened opposite the inner cell mass and blastocysts were incubated for a further 2 hours (approximately) to allow TE cell herniation as described previously (Kokkali et al. 2007). At the time of biopsy, blastocysts were placed individually in a dish prepared with 3 droplets of 10 µl of Quinn’s advantage medium enriched with HEPES and 5% HSA (SAGE, Trumbull, USA), overlaid with pre-equilibrated mineral oil for tissue culture (SAGE, Trumbull, USA) on a heated stage of an Olympus IX71 microscope, equipped with micromanipulation tools and a diode Laser (LYKOS, Hamilton-Thorne Biosciences, Beverly, USA). Each blastocyst was positioned on the holding pipette and oriented in a way that the ICM was clearly visible and on the side opposite the biopsy pipette (Cook Medical, Bloomington, USA). The TE cells (6-8) were gently aspirated with moderate suction into the biopsy pipette while synchronously firing 2 to 3 laser pulses aimed at the thinnest junctions between TE cells and stretching them gently to separate them from the blastocyst proper. Following the biopsy procedure, the blastocyst was placed in culture medium and incubated until the time of embryo-transfer. The retrieved biopsied TE cells were stored at -80°C until further use.

4. Clinical outcomes

Embryo implantation was determined by serum beta hCG levels (≥25 mIU/ml) (Roche Diagnostics International, Switzerland) on Day 16 after oocyte retrieval and confirmed by demonstration of a gestational sac by ultrasound scan, performed 4 weeks after embryo transfer. Clinical pregnancy was defined as the presence of a fetal heartbeat beyond 7 weeks of gestation.

5. RNA isolation and TE cDNA library construction

The non-stranded SMART-Seq v4 Ultra Low Input RNA Kit (Clontech, USA) was employed for the RNA library construction, according to the manufacturer's instructions. In brief, the biopsied TE cells from both competent and non-competent blastocyst groups were immediately transferred into the supplier's lysis buffer with the addition of RNase Inhibitor (Clontech, USA) and frozen at -80°C until required. First strand cDNA was optimised using the SMART-Seq locked nucleic acid (LNA) technology and the full cDNA length amplified by long-distance PCR (LD-PCR). Following optimization, 13 PCR cycles were selected. The same number of PCR cycles were applied to all samples, so that any PCR bias was minimized and the samples were comparable with the minimum introduced technical errors. Amplified cDNA was then purified using Agencourt Ampure XP Beads (Beckman Coulter, USA).

A Qubit dsDNA high sensitivity fluorometric assay was employed to quantify the amplified full-length cDNA (Thermo Fisher Scientific, USA). Approximately 150pg of this cDNA was further processed using Illumina’s Nextera XT DNA library preparation kit, according to the manufacturer’s instructions (Illumina, USA). The ‘tagmentation’ step of the full-length transcripts was followed by a 12-cycle PCR amplification step, using a unique combination of index 1 (i7) and index 2 (i5) adapters per library, according to the manufacturer instructions (Illumina, USA). The average length distribution of the fragmented cDNA libraries was assessed by the high sensitivity DNA kit (Agilent, USA) and the libraries quantified by Qubit (Thermo Scientific, USA), prior to equimolar pooling. The samples were sequenced on a HiSeq 3000 (Illumina, USA).

6. Bioinformatics Analysis

Sequence qualities were assessed using FastQC (Andrews 2010) and Trim galore! (Krueger 2015) was employed for automated Nextera adapter and quality trimming. Reads passing QC were mapped to the human reference genome (hg38) using the HISAT2 aligner version 2.0.4 (Pertea et al. 2016). Samtools version 1.3 was employed to remove unmapped and unpaired reads (Li et al. 2009), while PCR duplicates were tagged by Picard tools version 2.1.1 (Broad Institute. (2010). Available online at http://broadinstitute.github.io/picard). Binary files for the visualization of read coverage and depth on the UCSC Genome Browser (Kent et al. 2002) were generated with Bedtools version 2.25.0 (Quinlan and Hall 2010) and the bedGraphToBigWig UCSC utility (UCSCutilities Accessed on 2018). We used StringTie to detect potentially novel transcripts using the hg38 annotation file (UCSC) of the human transcriptome as a guide (Pertea et al. 2015). Two approaches were used to assign and quantify reads mapping to known and predicted transcripts to cross-check the robustness of differential gene expression (DE) detection. The first used a python script (PythonScript Accessed on March 2018), to extract read counts from the HISAT2/StringTie outputs in a format suitable as input for the Bioconductor/R package edgeR (Robinson and Oshlack 2010; Team 2013). The other used the featureCounts function of Rsubread to generate count tables (Liao et al. 2013). Only uniquely mapped, correctly paired and non-duplicated reads were considered in this study.

7. Statistical Analysis

The calcNormFactors function was used to normalize count data, based on the trimmed mean M value (TMM) (Robinson and Oshlack 2010). The thresholds for transcript inclusion in the DE analysis were at least 1 counts-per-million (cpm) reads present in at least 4 of the libraries. Transcripts represented at significantly different levels between successful and unsuccessful blastocysts were detected using edgeR with FDR set at 0.05 (Robinson and Oshlack 2010).

8. Gene networks, molecular interactions and ontological analysis

Gene ontology of all the detected transcripts, as well as the significantly up-regulated transcripts either in competent or incompetent blastocysts, were explored using DAVID (Huang et al. 2009). FDR/Benjamini–Hochberg procedure employed for multiple comparisons correction. DE transcripts were also submitted to the KEGG pathway database to determine the metabolic pathways involved in the successful blastocysts (Kanehisa et al. 2015). Ontological analysis of the TE transcripts was conducted using the BiNGO plugin (v3.0.3) through the Cytoscape platform (v3.3.0), revealing the metabolic pathways underlying TE gene expression (Shannon et al. 2003; Maere et al. 2005).

9. NGS validation using qRT-PCR

Validatory relative quantitative real-time PCR (qRT-PCR) was conducted on the synthesized cDNA followed by long distance PCR (LD PCR) enrichment, using an HT7900 thermal cycler (Applied Biosystems, USA). The annealing temperature per primer pair was set at 60°C (Supplemental Table 5). As we were solely concerned with confirming results of the Edge-R differential gene expression analysis, all PCR data for genes of interest were normalized against GAPDH using Livak’s method (Livak and Schmittgen 2001). To confirm primer specificity, amplicon sizes were checked by gel electrophoresis (Supplemental Figure 3; Supplemental Table 5).

10. SNP validation of blastocyst ploidy

We designed a workflow to process correctly paired, mapped and oriented HISAT2 alignments to generate variant calls from the mapped reads. Potential PCR duplicates were removed with Picard MarkDuplicates and read group information was added with Picard AddOrReplaceReadGroups (both accessible at https://broadinstitute.github.io/picard/). The GATK (v.4.1.0.0) tools SplitNCigarReads and HaplotypeCaller were used to create files in vcf.gz format for downstream analyses adapted from the eSNPKaryotyping protocol described by Weissbein (Weissbein et al. 2016). We used snp151Common table downloaded from the UCSC TableBrowser as a reference. The complete workflow and scripts used in the analysis are available for download from https://github.com/daveiles/human_TEbiopsy_eSNPanalysis.

**References**

Andrews S. 2010. FastQC: a quality control tool for high throughput sequence data. Available online at: http://www.bioinformatics.babraham.ac.uk/projects/fastqc.

Balaban B, Brison D, Calderón G, Catt J, Conaghan J, Cowan L, Ebner T, Gardner D, Hardarson T, Lundin K. 2011. The Istanbul consensus workshop on embryo assessment: proceedings of an expert meeting. Human Reproduction. 26(6):1270-1283.

Huang DW, Sherman BT, Lempicki RA. 2009. Bioinformatics enrichment tools: paths toward the comprehensive functional analysis of large gene lists. Nucleic acids research. 37(1):1-13.

Kanehisa M, Sato Y, Kawashima M, Furumichi M, Tanabe M. 2015. KEGG as a reference resource for gene and protein annotation. Nucleic acids research.gkv1070.

Kent WJ, Sugnet CW, Furey TS, Roskin KM, Pringle TH, Zahler AM, Haussler D. 2002. The human genome browser at UCSC. Genome research. 12(6):996-1006.

Kokkali G, Traeger-Synodinos J, Vrettou C, Stavrou D, Jones G, Cram D, Makrakis E, Trounson A, Kanavakis E, Pantos K. 2007. Blastocyst biopsy versus cleavage stage biopsy and blastocyst transfer for preimplantation genetic diagnosis of β-thalassaemia: a pilot study. Human Reproduction. 22(5):1443-1449.

mKrueger F. 2015. Trim Galore!: A wrapper tool around Cutadapt and FastQC to consistently apply quality and adapter trimming to FastQ files.

Li H, Handsaker B, Wysoker A, Fennell T, Ruan J, Homer N, Marth G, Abecasis G, Durbin R. 2009. The sequence alignment/map format and SAMtools. Bioinformatics. 25(16):2078-2079.

Liao Y, Smyth GK, Shi W. 2013. The Subread aligner: fast, accurate and scalable read mapping by seed-and-vote. Nucleic acids research. 41(10):e108-e108

.

Livak KJ, Schmittgen TD. 2001. Analysis of relative gene expression data using real-time quantitative PCR and the 2− ΔΔCT method. Methods. 25(4):402-408.

Maere S, Heymans K, Kuiper M. 2005. BiNGO: a Cytoscape plugin to assess overrepresentation of gene ontology categories in biological networks. Bioinformatics. 21(16):3448-3449.

Pertea M, Kim D, Pertea GM, Leek JT, Salzberg SL. 2016. Transcript-level expression analysis of RNA-seq experiments with HISAT, StringTie and Ballgown. Nature Protocols. 11(9):1650-1667.

Pertea M, Pertea GM, Antonescu CM, Chang T-C, Mendell JT, Salzberg SL. 2015. StringTie enables improved reconstruction of a transcriptome from RNA-seq reads. Nature biotechnology. 33(3):290. PythonScript. Accessed on March 2018. prepDE.py. https://ccbjhuedu/software/stringtie/dl/prepDEpy.

Quinlan AR, Hall IM. 2010. BEDTools: a flexible suite of utilities for comparing genomic features. Bioinformatics. 26(6):841-842.

Robinson MD, Oshlack A. 2010. A scaling normalization method for differential expression analysis of RNA-seq data. Genome biology. 11(3):R25.

Shannon P, Markiel A, Ozier O, Baliga NS, Wang JT, Ramage D, Amin N, Schwikowski B, Ideker T. 2003. Cytoscape: a software environment for integrated models of biomolecular interaction networks. Genome research. 13(11):2498-2504.

Team RC. 2013. R: A language and environment for statistical computing. R Foundation for Statistical Computing, Vienna, Austria.[WWW document]. URL http://www R-project org/[Accessed December 24, 2013].

UCSCutilities. Accessed on 2018.

http://hgdownloadsoeucscedu/downloadshtml#utilities_downloads.

Weissbein U, Schachter M, Egli D, Benvenisty N. 2016. Analysis of chromosomal aberrations and recombination by allelic bias in RNA-Seq. Nature communications. 7:12144.
