# Supplementary material for: Can trophectoderm RNA analysis predict human blastocyst competency?
Source: Syst Biol Reprod Med. 2019 Jun 27;65(4):312–25. doi: 10.1080/19396368.2019.1625085 (PMC6816490; doi:10.1080/19396368.2019.1625085)
Supplement: Supplemental Material [file IAAN_A_1625085_SM9021.zip › Ntostis_etal_supplemental_Table_5.docx]

**Supplemental Table 5. List of DE transcripts and primers selected to confirm the RNA sequencing results**.

| **Gene Target** | **Forward** | **Length** | **Reverse** | **Length** | **T anneal (°C)** | **Amplicon (bp)** |
| --- | --- | --- | --- | --- | --- | --- |
| GAPDH | TTGTCAAGCTCATTTCCTGGTAT | 23 | TCTCTCTTCCTCTTGTGCTCTTG | 23 | 60 | 135 |
| HSD17B1 | AGCTTCAAAGTGTATGCCACG | 21 | TGAGTCCCTTACGTCCAGC | 19 | 60 | 129 |
| CYP11A1 | CCGTGACCCTGCAGAGATAT | 20 | AAGAAGAAGGTGGGCTCTCG | 20 | 60 | 118 |
| DHCR7 | CTGGACCCTCATCAACCTGT | 20 | AGGTACCAGGTTTCGTTCCA | 20 | 60 | 135 |
| BAK1 | TTCACCAAGATTGCCACCAG | 20 | ATGCTGGTAGACGTGTAGGG | 20 | 60 | 105 |
| KHDC1P1 | GGTACGCTAAGACAGACGGT | 20 | TCGTCACAATCAAGGCCTCA | 20 | 60 | 150 |
