# Supplementary material for: Can trophectoderm RNA analysis predict human blastocyst competency?
Source: Syst Biol Reprod Med. 2019 Jun 27;65(4):312–25. doi: 10.1080/19396368.2019.1625085 (PMC6816490; doi:10.1080/19396368.2019.1625085)
Supplement: Supplemental Material [file IAAN_A_1625085_SM9021.zip › Upload_2018_336.r2_Supplemental_table_titles_and_info_material (5).docx]

**Supplemental Table 1. Full list of TE transcripts identified by RNA sequencing and StringTie.**

All transcripts meeting the criteria of ≥1.0 cpm in at least four TE biopsies are listed. The eight blastocysts include four that were competent (COMP 1-4) and four that either failed to implant or did not support a clinical pregnancy (INCOMP 1-4). Apart from known genes indicated by their refseq ID, there are also StringTie assembled reads to novel not annotated transcripts.

**Supplemental Table 2.** **Ontological analysis** **of all TE transcripts**.

Biological Process terms (DAVID) for transcripts with expression levels exceeding 1.0 cpm are shown. The list is sorted by FDR (from low to high).

**Supplemental Table 3. Biological processes and functional annotation clustering (FAC) analysis of DE transcripts**.

Tab 1 (BP_UNS_DWN) depicts lists of transcripts significantly down-regulated in incompetent (INCOMP) blastocysts. Tabs 2 (FAC_UNS_DOWN) and 3 (FAC_UNS_UP) represent the functional annotation clustering (FAC) of the down-regulated and up-regulated transcripts in the INCOMP blastocysts, respectively. The lists are sorted by FDR (low to high) and enrichment score (high to low) for the FAC.

**Supplemental Table 4. Common gene family members between the DE genes reported in the current and Kirkegaard analysis.**

Gene family names are shown alongside their respective gene family members for both studies.

**Supplemental Table 5. List of DE transcripts and primers selected to confirm the RNA sequencing results**.

The table includes the PCR product sizes (see supplemental figure 3 for associated agarose gel).
