# Supplementary figures and images for: Can trophectoderm RNA analysis predict human blastocyst competency?
Source: Syst Biol Reprod Med. 2019 Jun 27;65(4):312–25. doi: 10.1080/19396368.2019.1625085 (PMC6816490; doi:10.1080/19396368.2019.1625085)

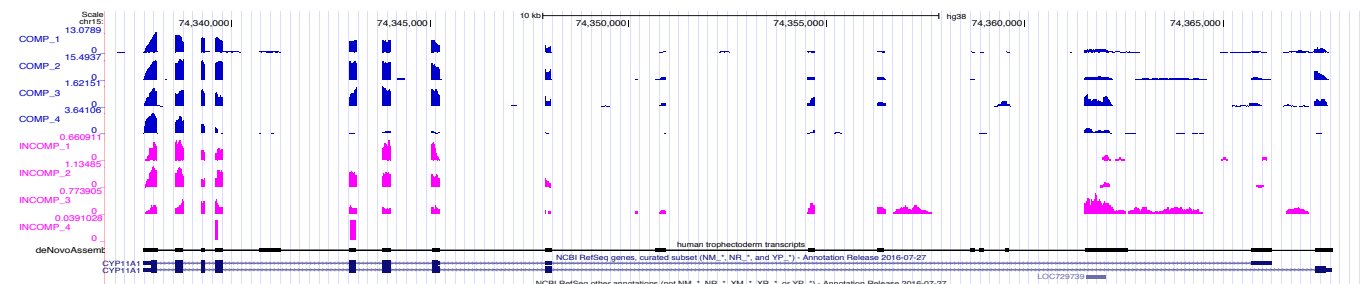

A: CYP11A1

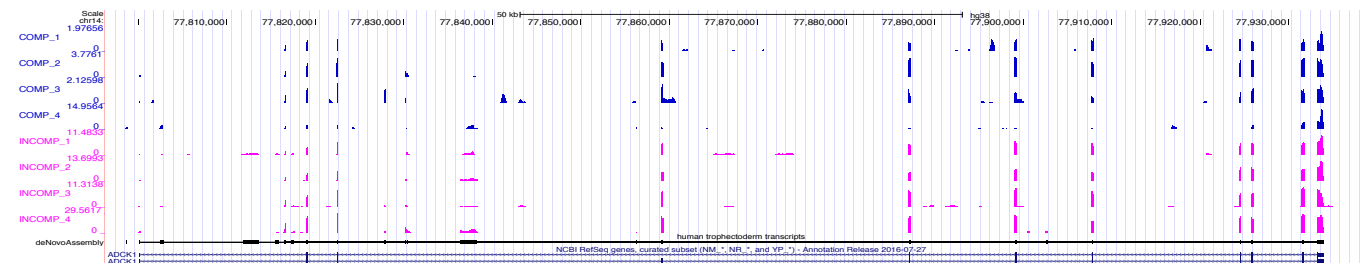

B: ADCK1

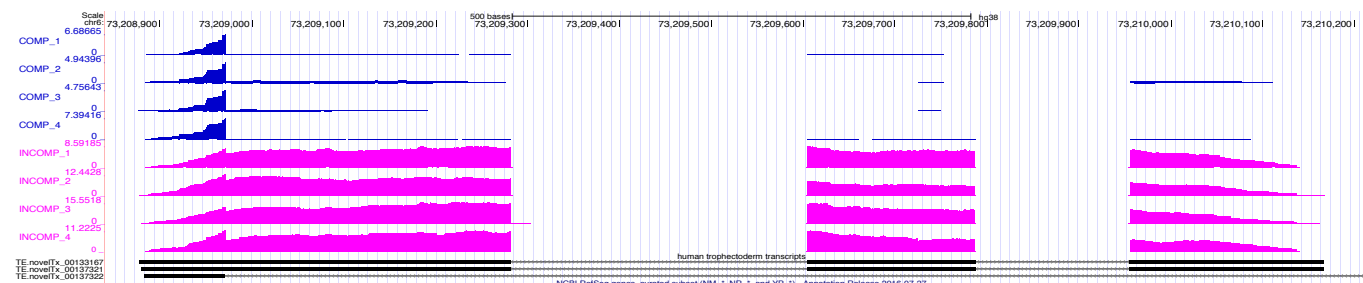

C: KHDC1P1

Supplement: Supplemental Material [file IAAN_A_1625085_SM9021.zip › Ntostis_etal_suppl_Figure1.pdf]

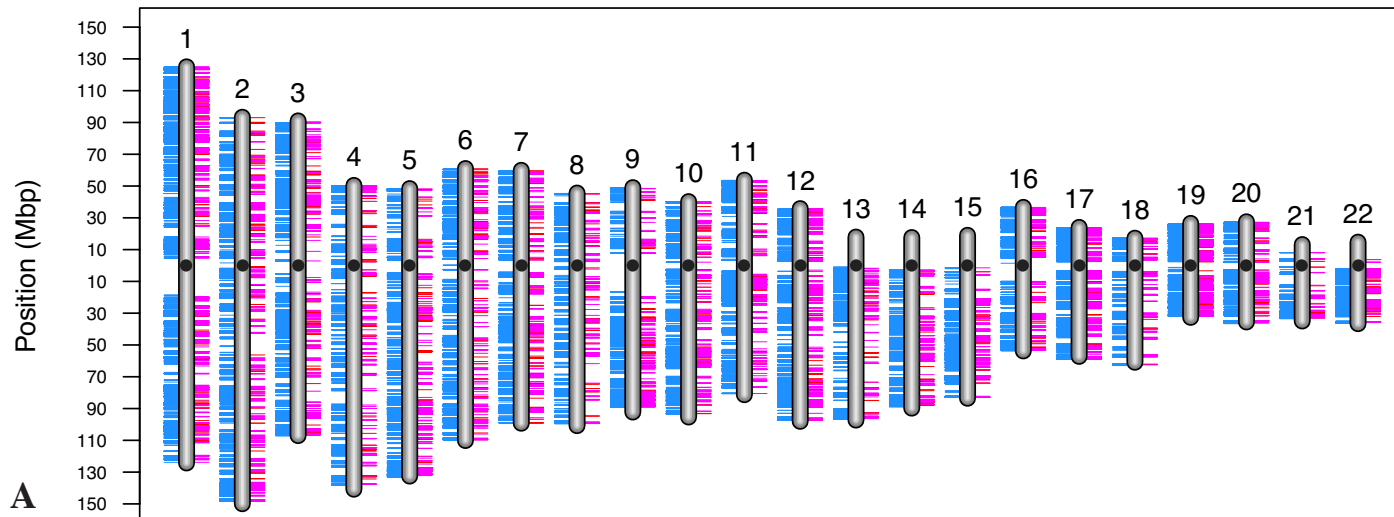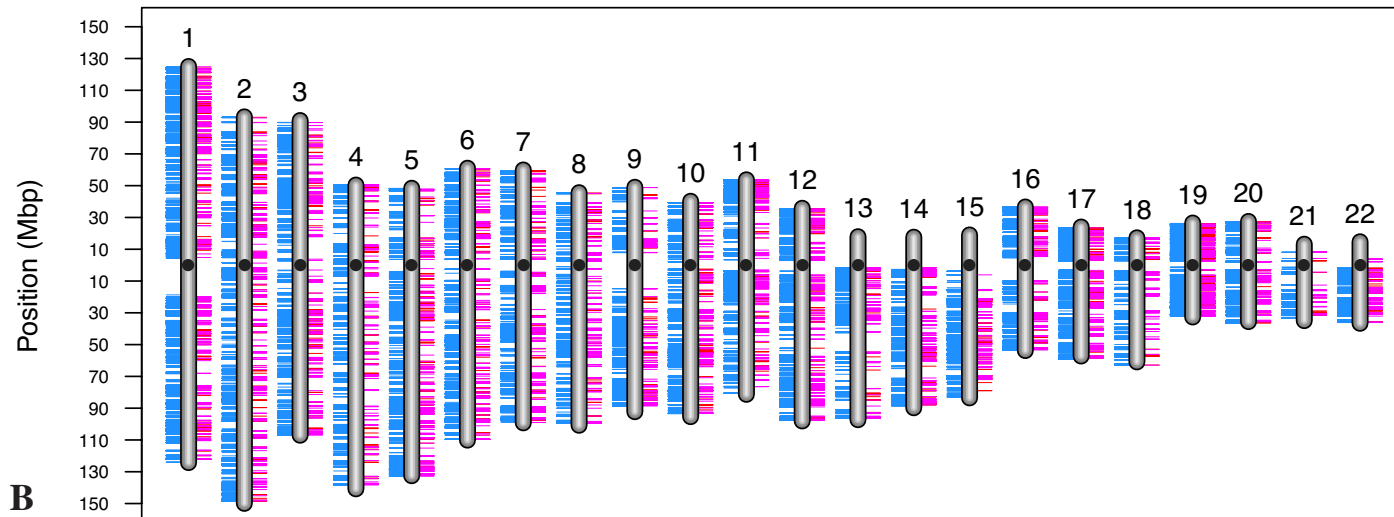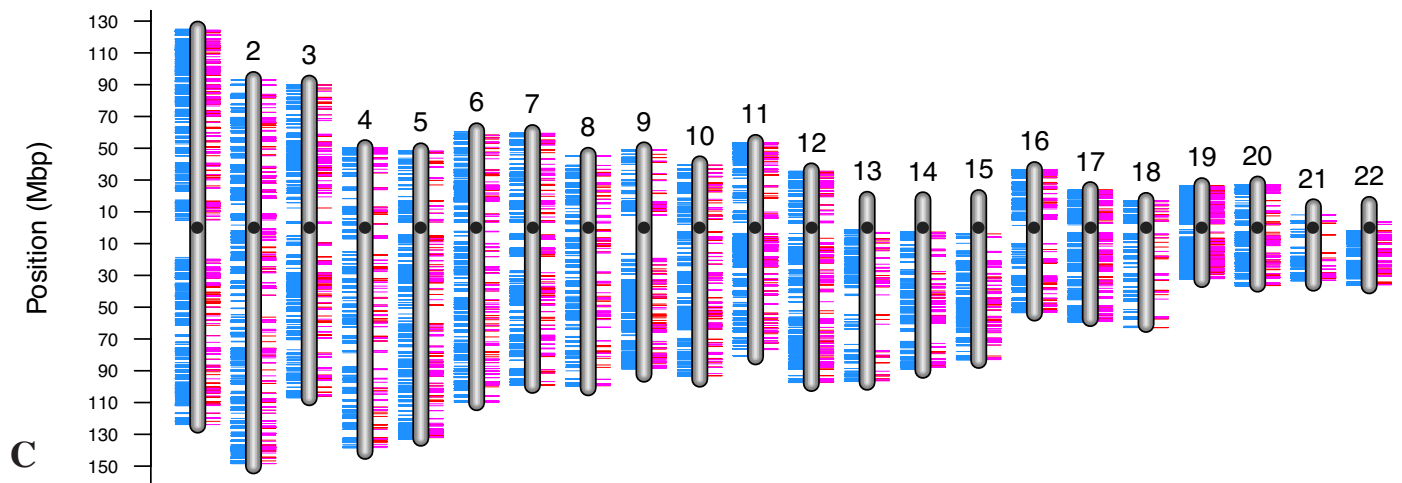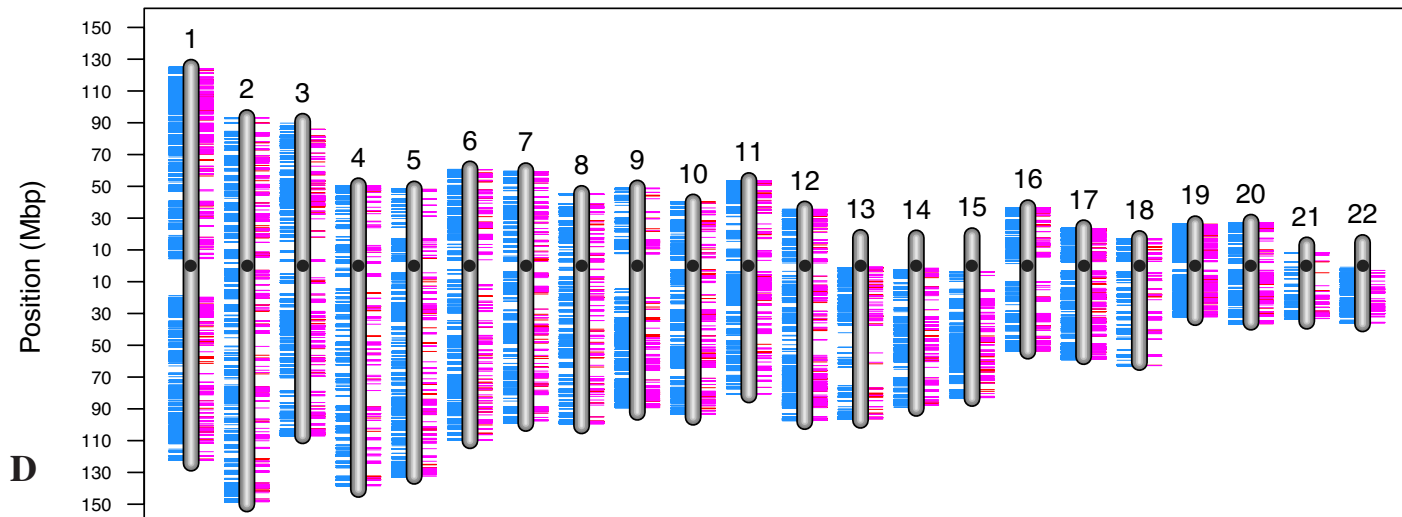

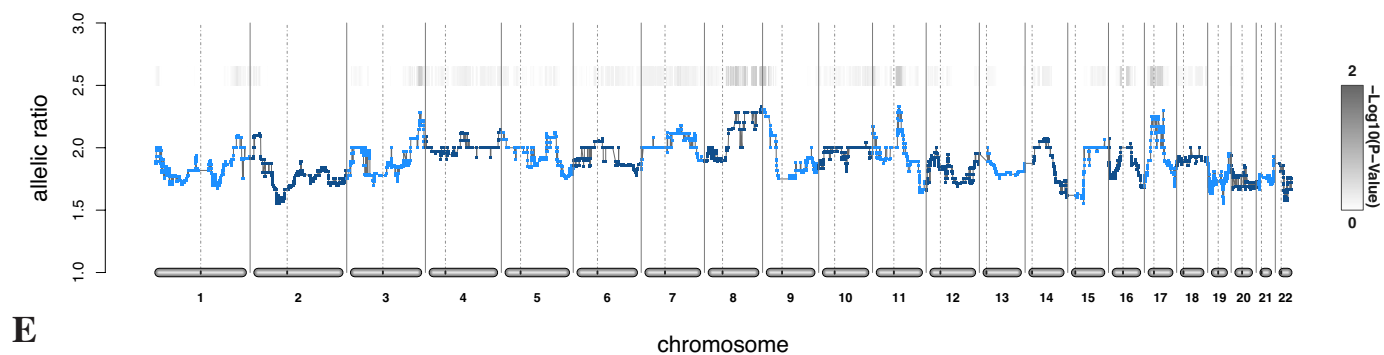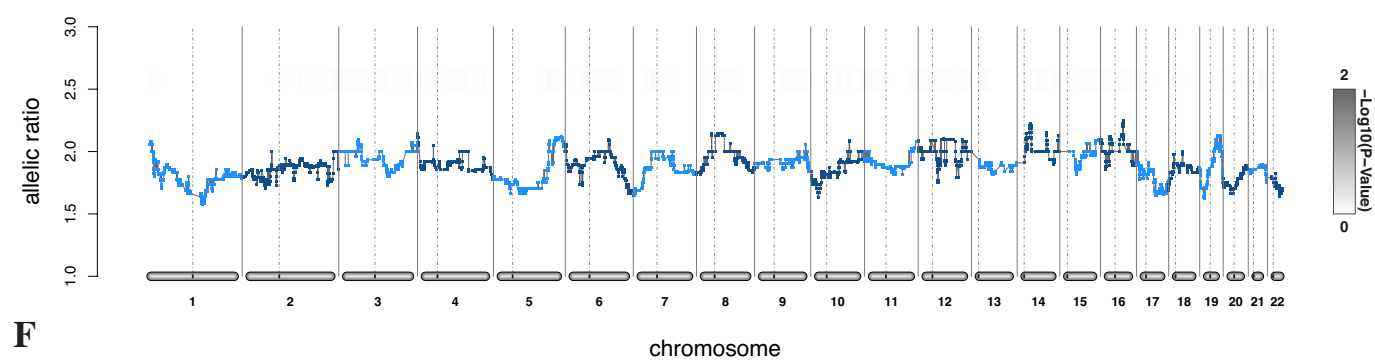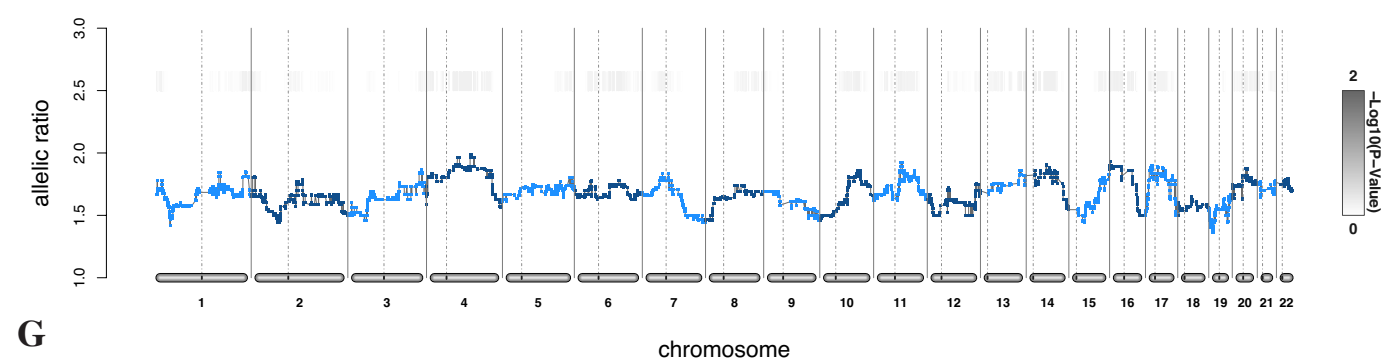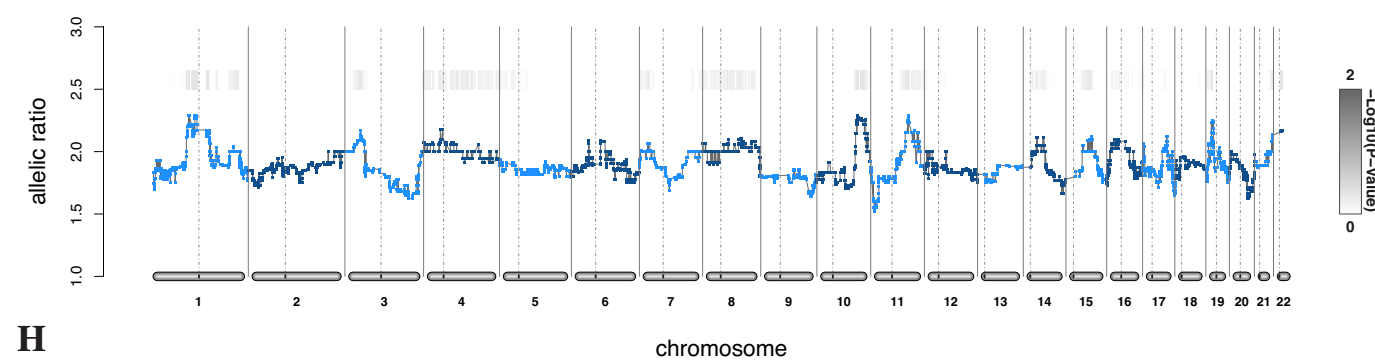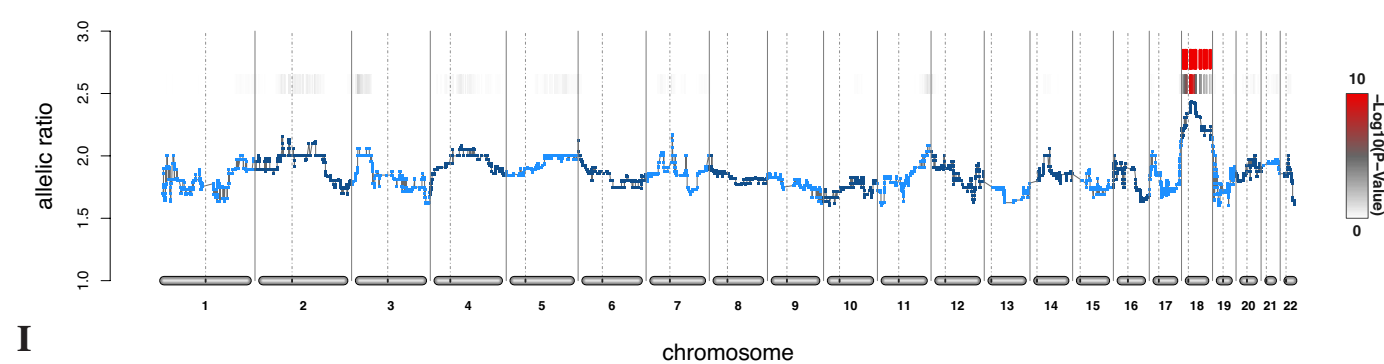

Supplement: Supplemental Material [file IAAN_A_1625085_SM9021.zip › Ntostis_etal_supplemental_Figure_2.pdf]

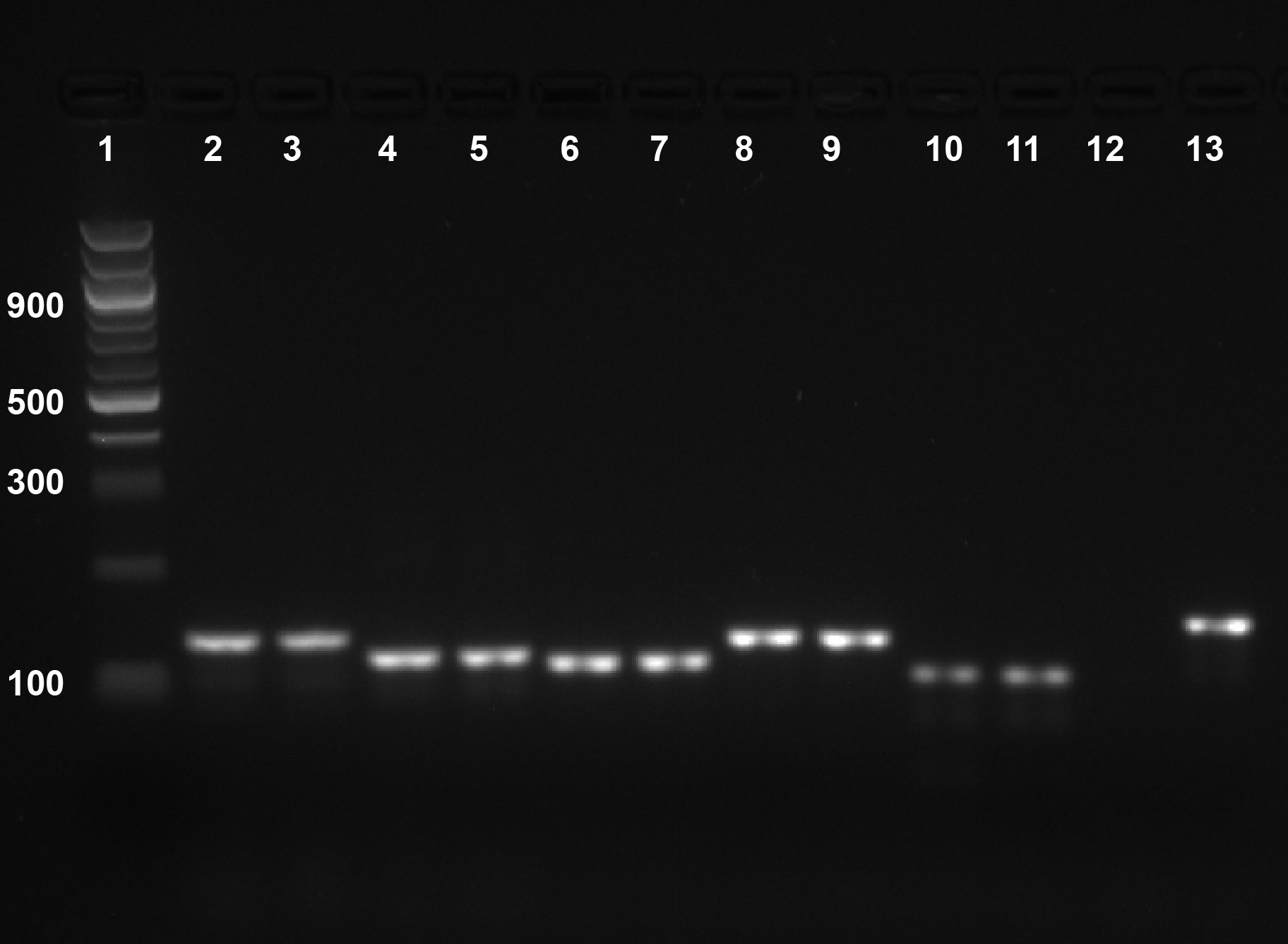

Supplement: Supplemental Material [file IAAN_A_1625085_SM9021.zip › Ntostis_etal_supplemental_Figure_3.jpg]
